# Characteristics of Obstructive Sleep Apnea Across the Spectrum of Glucose Tolerance in Obese Adolescents

**DOI:** 10.3389/fendo.2018.00281

**Published:** 2018-06-01

**Authors:** Tamara S. Hannon, Sara E. Watson, Hasnaa E. Jalou, Sangeeta Chakravorty, Kieren J. Mather, Silva A. Arslanian

**Affiliations:** ^1^Department of Pediatrics, Indiana University School of Medicine, Indianapolis, IN, United States; ^2^Department of Pediatrics, University of Louisville School of Medicine, Louisville, KY, United States; ^3^Department of Pediatrics, University of Pittsburgh Medical Center, Pittsburgh, PA, United States; ^4^Department of Medicine, Indiana University School of Medicine, Indianapolis, IN, United States

**Keywords:** obesity, prediabetes, pediatric, sleep-disordered breathing, apnea, insulin sensitivity

## Abstract

**Background:**

It is not known if dysglycemia and sleep-disordered breathing are linked in adolescents, as in adults.

**Objective:**

To perform a pilot study evaluating measures of sleep-disordered breathing across the spectrum of glucose tolerance in obese adolescents. We hypothesized that dysglycemia would be associated with sleep-disordered breathing.

**Participants/methods:**

This was a prospective, cross-sectional clinical pilot study that included 57 adolescents [body mass index (BMI) 38.9 ± 8.4 kg/m^2^] aged 12–18 years (14.5 ± 1.6) with normal glucose tolerance (NGT), or dysglycemia [impaired glucose tolerance (IGT) or type 2 diabetes (T2D)].

**Measures:**

Anthropometrics, overnight polysomnogram, and oral glucose tolerance tests were performed. Participant characteristics and outcome measures were compared by glucose tolerance status. Correlational analyses were conducted to assess the associations between variables of interest.

**Results:**

Participants with dysglycemia (*n* = 21) were not different from those with NGT (*n* = 36) for BMI, waist circumference, body fat, or sleep characteristics. Nocturnal oxygen desaturation was associated with higher BMI (*r* = −0.334, *p* = 0.012). The apnea–hypopnea index (AHI) was not associated with physical and metabolic parameters. Although participants with dysglycemia tended to have higher AHIs (median 3.2, 2.2, and 1.6 events/h for T2D, IGT, and NGT, respectively), there was not a linear relationship between measures of glycemia and AHI.

**Conclusion:**

Further study with a larger proportion of youth with prediabetes and T2D is necessary to determine whether evaluation for sleep-disordered breathing is uniformly warranted.

## Introduction

Prediabetes and type 2 diabetes (T2D) are strongly associated with obstructive sleep apnea (OSA) in adults (1). In the Look AHEAD study, >80% of overweight adult participants with T2D were found to have OSA on screening polysomnogram (PSG) (2). In adults, OSA is linked with higher hemoglobin A1C values across the range of glucose tolerance (3), and treatment of OSA has been associated with improvement in blood glucose control (4, 5). In youth, it is unknown if prediabetes and/or T2D and OSA or sleep-disordered breathing are linked, as few similar studies have been performed (6). In this pilot study, we sought to evaluate if sleep-disordered breathing is prevalent in obese adolescents with dysglycemia, and whether sleep-disordered breathing is associated with blood glucose measures. This is important to study in adolescents because: (1) adolescents have physiologic insulin resistance which increases risk for dysglycemia and development of prediabetes and T2D (7); (2) adolescents with obesity and T2D have particularly poor health outcomes (8, 9); and (3) longer exposure to T2D and sleep-disordered breathing may further exacerbate these poor health outcomes.

The primary objective of this pilot study was (a) to evaluate PSG-derived measures of sleep-disordered breathing across the spectrum of glucose tolerance in obese adolescents. We utilized oral glucose tolerance tests (OGTT) to categorize youth as having normal glucose tolerance (NGT), or dysglycemia [OGTT values consistent with prediabetes defined as impaired fasting glucose (IFG) and/or impaired glucose tolerance (IGT), and T2D], and (b) to examine the associations between OGTT-derived measures of glucose tolerance, insulin sensitivity, and insulin secretion and PSG-derived measures of OSA. We hypothesized that obese youth with dysglycemia would have more characteristics of OSA than obese youth with NGT.

## Materials and Methods

This cross-sectional study was approved by the University of Pittsburgh and Indiana University Institutional Review Boards and performed in the Pediatric Clinical and Translational Research Center (Children’s Hospital of Pittsburgh) and the Indiana Clinical Research Center with collaboration from the clinical sleep centers at these institutions. Informed and written consent was obtained from a parent/guardian, and assent was obtained from participants. A convenience sample of eligible subjects referred to outpatient weight management or endocrinology clinics for obesity, prediabetes, or T2D, and who also reported snoring were approached for participation. Eligibility criteria were 12- to 18-year-old male or female adolescents (Tanner stage >1) of any race with body mass index (BMI) ≥95th percentile. Exclusion criteria included diagnosis of type 1 diabetes, current tonsillar hypertrophy, chronic disease, or medications that may interfere with sleep, endocrine function or glucose regulation, syndromic obesity, chronic upper or lower airway disease, smoking, or current upper respiratory tract infection. Participants treated with either metformin and/or lifestyle recommendations were eligible, after discontinuing metformin/exercise treatment for 48 h before study. If there was a previous diagnosis of T2D, duration was less than 2 years for all participants. Ninety-six patients were screened for the study, and 57 participants enrolled and completed study visits after informed consent/assent was obtained. Data for part of this study cohort have been previously published (10–12).

### Study Procedures and Assays

All participants had an examination by a pediatric endocrinologist that included assessment of pubertal development, anthropometric measurements, and 53/57 had body composition by dual-energy X-ray absorptiometry (4 had body weights greater than machine limits). A fasting laboratory evaluation including HbA1C, glucose, and insulin, and 2-h OGTT (1.75 g/kg, maximum 75 g; glucose and insulin at −15, 0, +15, +30, +60, +90, and +120 min time points) was performed. Fifty-three of fifty-seven participants completed the OGTT without complications. The remaining participants had minor complications, usually related to loss of intravenous access, which prohibited frequent blood sampling or reduced sample volume. Plasma glucose was measured by the glucose oxidase method (Pittsburg) and the glucose hexokinase method (Indianapolis). Samples were re-measured in Indianapolis using the glucose hexokinase method when available. Plasma insulin was determined by Beckman Coulter DXI 800 using a chemiluminescent sandwich assay (CV 6%).

### Polysomnograms

Overnight PSGs were performed in the sleep lab either the night immediately preceding the OGTT or the night immediately following the OGTT. Due to scheduling issues, it was not possible to conduct all studies in the same order. There were no differences in outcomes based on timing of the OGTT. Data were recorded using either Sensormedics Somnostar Pro version 7.2 or the Sandman Elite 9.1 sleep diagnostic software, and applying the following EEG montage: F3M2, F4M1, C3M2, C4M1, O2M1, O1M2, L-EOG, R-EOG, chin EMG, limb EMG, and the following cardiorespiratory parameters: SpO_2_ and pulse (Masimo), ETCO2 (Microstream NPB 70 and Capnograph Sleep by BCI), nasal pressure, airflow (nasal or oral thermistor), thoracic and abdominal excursion (uncalibrated respirator inductance plethysmography), pulse, and ECG. The PSG data were interpreted by one of two sleep Board-certified co-investigators specific to the site where the PSG was performed, who had no knowledge of the participants’ metabolic status. Sleep architecture and respiratory disturbances including the minimum percent oxyhemoglobin saturation (SpO_2_) by pulse oximetry, arousal events per hour (sleep fragmentation), and the apnea–hypopnea index (AHI, total number of central and obstructive apnea and hypopnea events per 1 h of sleep), were hand-scored utilizing pediatric criteria and calculated following the AASM manual for scoring guidelines (13). Obstructive apneas were scored for a drop in the peak flow signal by ≥90% of the pre-event baseline using an oral thermal sensor lasting ≥90% for the duration of at least two breaths during baseline breathing and associated with the presence of respiratory efforts throughout the entire period of the absent flow. Hypopneas were scored for a drop by ≥30% of the pre-event baseline using nasal pressure lasting ≥two breaths duration in association with either ≥3% oxygen desaturation from the pre-event baseline or the event is associated with an arousal. Nocturnal hypoxia is defined as the time spent with SpO_2_ less than 90% for more than 5 min.

### Calculations

Insulin sensitivity was estimated using inverse fasting insulin (14). Insulin secretion was expressed as the ratio of the incremental response of insulin to glucose at 30 min during the OGTT [insulinogenic index (IGI), ΔI_0–30_/ΔG_0–30_], i.e., the IGI (15). Insulin secretion in relation to insulin sensitivity (oral disposition index) was calculated as (ΔI_0–30_/ΔG_0–30_ × 1/fasting insulin) (15). OGTT glucose and insulin areas under the curve (AUC) were calculated using the trapezoidal method.

### Statistical Analysis

Demographic and clinical characteristics of the cohort were summarized, and participants were compared by glucose tolerance status [NGT or having dysglycemia (IFG, IGT, or T2D)]. Dysglycemia was defined as having IFG or IGT using American Diabetes Association (ADA) criteria (16). T2D was defined using ADA criteria for OGTT results (16). Data were log-transformed when not normally distributed. Scatter plots were used to examine the relationships between variables of interest for the entire study cohort, and for NGT and dysglycemia groups. Non-parametric tests for independent samples were performed using Kruskal–Wallis one-way ANOVA. Spearman’s correlation coefficients were used to quantify linear correlations, as data were not uniformly distributed for the cohort. Given the pilot nature of the study, we did not perform a pre-study sample-size calculation to address whether or not OSA was related to prediabetes or T2D in adolescent youth. *Post hoc* power analysis showed that with the current sample size, we had 80% power to detect correlation coefficients greater than 0.37 (17). Considering the preliminary nature of the investigation, we did not perform multiplicity adjustment. *p* Values < 0.05 were considered statistically significant. All analyses were implemented using SPSS software (Version 24).

## Results

Characteristics of the participants are reported in Table 1. Thirty-six (63.2%) had OGTT results consistent with NGT; 21 had dysglycemia on OGTT [IFG (*n* = 5), IGT (*n* = 11), both IFG and IGT (*n* = 3), FPG ≥126 mg/dL (*n* = 3), OGTT 2-h glucose ≥200 mg/dL (*n* = 5)]. Participants with dysglycemia were not significantly different from those with NGT for BMI, BMI SDS, or waist circumference. Percent body fat was higher in the group with dysglycemia (Table 1). Sleep characteristics among the groups divided by glycemia categories are shown in Table 1. There were no sex- or race-related differences in sleep characteristics. Seventeen (29.8%) had normal sleep studies with AHI < 1; 24 (42.1%) had AHI ≥1 to <5; 8 (14.0%) had AHI ≥5 to <10; 6 (10.5%) had AHI ≥10 to <15; 2 (3.5%) had AHI ≥15.

**Table 1:** Characteristics of the study participants.

	Total study population	Normoglycemic	Dysglycemic	*p* Value
Total (%)	57 (100)	36 (63.2)	21 (36.8)	
Age, years (SD)	14.5 ± 1.6	14.5 ± 1.6	14.2 ± 1.7	0.50
Sex				
Female	30 (52.6%)	15 (41.7%)	15 (71.4%)	0.03
Male	27 (47.4%)	21 (53.3%)	6 (28.6%)	
Race				
White	29 (50.9%)	19 (52.8%)	10 (47.6%)	0.66
Black	27 (47.4%)	16 (44.4%)	11 (52.4%)	
Mixed race	1 (1.8%)	1 (2.8%)	0 (0%)	
Body mass index (BMI) (kg/m^2^)	38.9 ± 8.4 (35.0, 28.4–61.6)	38.3 ± 8.4 (34.8, 28.4–61.6)	39.8 ± 8.5 (35.5, 28.7–57.1)	0.53
BMI SDS	3.88 ± 1.50 (3.43, 1.80–8.31)	3.83 ± 1.57 (3.35, 1.80–8.31)	3.95 ± 1.39 (3.80, 2.04–6.43)	0.77
Waist circumference (cm)	113.8 ± 17.8 (109.0, 78.1–155.0)	115.3 ± 16.6 (111.6, 91.4–155.0)	111.1 ± 19.8 (106.0, 78.1–149.0)	0.39
Body fat (%)	46.4 ± 6.1 (*n* = 53) (46.3, 36.7–58.5)	45.3 ± 5.7 (*n* = 32) (44.7, 36.8–58.5)	48.9 ± 6.1 (48.9, 36.7–57.2)	0.035

**Laboratory characteristics**				
HbA1c (%)	5.8 ± 1.3 (5.4, 4.5–12.0)	5.5 ± 0.8 (5.4, 4.5–9.6)	6.3 ± 1.8 (5.9, 5.0–12.0)	0.014
Fasting glucose (mg/dL)	98.3 ± 7.4 (91.5, 74–231)	88.6 ± 6.5 (89.3, 74–99)	114.9 ± 37.4 (101.0, 79–231)	<0.001
OGTT 2-h glucose (mg/dL)	143 ± 52 (*n* = 56) (128, 89–332)	118.8 ± 13.7 (122, 89–138)	187.2 ± 66.5 (157.5, 98–332)	<0.001
1/Fasting insulin (μU/mL)	0.0421 ± 0.0210 (*n* = 52) (0.0385, 0.0100–0.0900)	0.0481 ± 0.0210 (n = 34) (0.0446, 0.0200–0.0900)	0.0307 ± 0.0159 (*n* = 18) (0.0321, 0.0100–0.0700)	0.003
Insulinogenic index	3.79 ± 2.64 (*n* = 51) (3.01, 0.30–12.3)	3.60 ± 2.0 (*n* = 33) (3.27, 0.36–7.83)	4.14 ± 3.57 (*n* = 18) (2.63, 0.30–12.26)	0.95
Oral disposition index	13.6 ± 10.0 (*n* = 51) (11.8, 0.81–56.6)	15.4 ± 10.3 (*n* = 33) (13.9, 2.5–56.6)	10.2 ± 8.6 (*n* = 18) (7.0, 0.8–34.5)	0.022

**Sleep characteristics**				
Polysomnogram sleep time, min	411 ± 53 (415, 233–494)	406 ± 53 (415, 233–478)	419 ± 54 (414, 314–494)	0.61
Sleep latency, min	15.5 ± 14.4 (12.0, <1.0–67.0)	14.8 ± 13.6 (12.3, 1.0–67.0)	16.6 ± 16.0 (12.0, <1.0–54.0)	0.93
REM sleep, % of total	18.9 ± 5.2	18.9 ± 5.5	18.9 ± 4.6	0.99
Slow wave sleep, % of total	19.4 ± 7.1	20.0 ± 6.9	18.3 ± 7.6	0.42
Apnea–hypopnea Index (AHI), events/h	4.7 ± 8.1 (2.2, 0.0–50.4)	3.3 ± 4.1 (1.6, 0.0–20.0)	7.0 ± 11.0 (2.9, 0.0–50.4)	0.19
AHI <1	17 (29.8)	12 (33.3)	5 (23.8)	
Mild obstructive sleep apnea (OSA); AHI ≥1 to <5	24 (42.1)	17 (47.2)	7 (33.3)	
Moderate OSA; AHI ≥5 to <10	8 (14.0)	4 (11.1)	4 (19.0)	
Severe OSA; AHI ≥10	8 (14.0)	3 (8.3)	5 (23.8)	
Adult OSA cutoff met, AHI >5, *n* (%)	16 (28.1)	7 (19.4)	9 (42.9)	0.07^a^
Arousal index, events/h	9.7 ± 6.3 (8.4, 2.0–39.2)	9.4 ± 4.7 (8.7, 3.5–25.3)	10.3 ± 8.8 (7.9, 2.0–39.2)	0.67
Nadir SpO_2_, %	88.6 ± 6.5 (91, 58–96)	90.3 ± 4.7 (92, 77–96)	86.0 ± 8.4 (87.0, 58–94)	0.06

*Data are means ± SD unless otherwise indicated. Median, range are given in parentheses for variables not normally distributed*.

*^a^Fisher’s Exact Test for the proportion with AHI >5 in each group (normoglycemic and dysglycemic)*.

**Figure 1:**
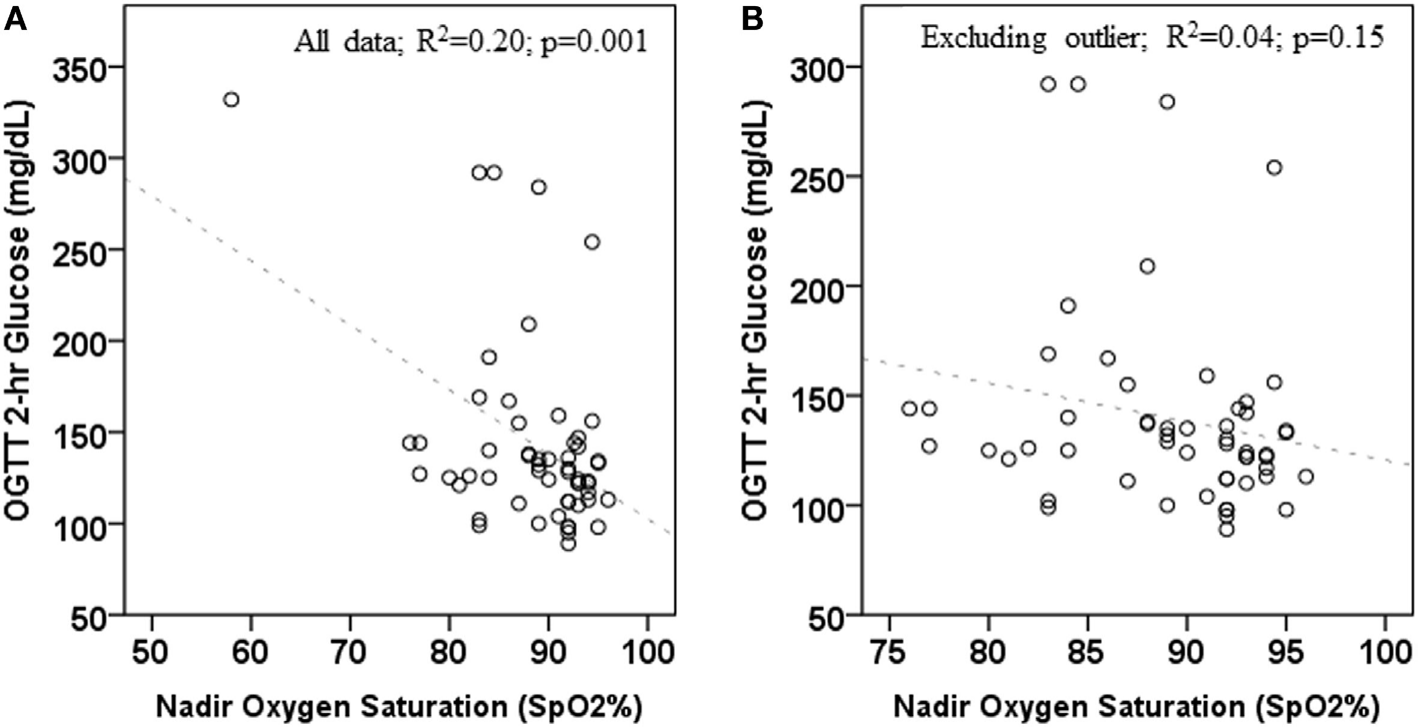
Association between 2-h oral glucose tolerance tests (OGTT) glucose concentration and nadir oxygen saturation during polysomnogram. **(A)** includes all data; **(B)** excludes one outlier.

The numbers and percentages of participants meeting criteria for OSA based on the AHI (mild, 1 to <5 events/h; moderate, 5 to <10 events/h; severe, ≥10 events/h) are shown in Table 1. The majority of participants had an AHI <5 events/h. The percentage of participants meeting criteria for any degree of OSA was 66.7% in those with normal glycemia and 76.2% in those with dysglycemia. The between-group difference was not statistically significant (*p* = 0.07). Spearman’s correlation coefficients for the associations between laboratory markers of glycemia and β-cell function, and indices of sleep fragmentation and sleep-disordered breathing (arousal events per hour, AHI, and nadir SpO_2_ during PSG) are shown in Table 2. BMI, waist circumference, percent body fat, and laboratory measures were not associated with the arousal index or the AHI. Higher BMI, higher waist circumference, and higher fasting and 2-h glucose concentrations were associated with lower nadir nocturnal SpO_2_. However, there was an outlier for nadir nocturnal SpO_2_ (58%, >2 SD from the mean). When the outlier was removed, the association between nadir nocturnal SpO_2_ and BMI remained (*r* = −0.334, *p* = 0.012), but the associations with waist circumference (*r* = −0.0258, *p* = 0.055), and with fasting (*r* = −0.229, *p* = 0.09) and 2-h glucose (*r* = −0.244, *p* = 0.07) were no longer significant (Figure 1).

**Table 2:** Correlation coefficients for the associations of sleep measures with physical and metabolic parameters.

	Body mass index	Waist circ.	Body fat (%)	HbA1c	Fasting glucose	2-h Glucose	1/Fasting insulin	Insulinogenic index	Oral disposition index
Arousal index, events/h	−0.074 (0.59)	−0.031 (0.82)	−0.164 (0.26)	0.151 (0.28)	−0.086 (0.54)	−0.074 (0.60)	0.044 (0.76)	−0.064 (0.66)	−0.094 (0.52)

Apnea–hypopnea index, events/h	0.175 (0.19)	0.169 (0.21)	−0.037 (0.79)	0.044 (0.74)	0.184 (0.17)	0.156 (0.25)	−0.089 (0.53)	−0.087 (0.55)	−0.177 (0.21)

Nadir SpO_2_, %	−0.318 (0.02)	−0.263 (0.048)	0.081 (0.56)	−0.087 (0.52)	−0.266 (0.046)	−0.284 (0.03)	0.239 (0.09)	−0.056 (0.69)	0.068 (0.64)

*Data are Spearman’s coefficients (*p* values)*.

When only participants with NGT were evaluated, lower nadir SpO_2_ was associated with higher BMI (*r* = − 0.34, *p* = 0.04), higher waist circumference (*r* = − 0.37, *p* = 0.03), lower inverse fasting insulin (*r* = 0.41, *p* = 0.02), and IGI (*r* = − 0.40, *p* = 0.02) (Table 3). There were no significant relationships between laboratory and sleep measures in the participants with dysglycemia (Table 4). However, among those with T2D, nadir SpO_2_ was significantly associated with waist circumference (*r* = − 0.86, *p* = 0.007).

**Table 3:** Correlation coefficients for the associations of sleep measures with physical and metabolic parameters, normal glucose tolerance group only.

	Body mass index	Waist circ.	Body fat (%)	HbA1c	Fasting glucose	2-h Glucose	Glucose areas under the curve	1/Fasting insulin	Insulinogenic index	Oral disposition index
Arousal index, events/h	−0.086 (0.74)	−0.104 (0.55)	0.062 (0.74)	0.303 (0.07)	0.034 (0.84)	−0.037 (0.83)	0.234 (0.18)	0.047 (0.79)	−0.236 (0.19)	−0.319 (0.07)

Apnea–hypopnea index, events/h	0.143 (0.41)	0.247 (0.15)	−0.064 (0.73)	−0.029 (0.87)	0.260 (0.13)	0.012 (0.95)	0.149 (0.40)	−0.198 (0.26)	−0.087 (0.55)	−0.177 (0.21)

Nadir SpO_2_, %	−0.342 (0.04)	−0.372 (0.03)	0.151 (0.40)	−0.010 (0.95)	−0.283 (0.10)	−0.127 (0.46)	−0.064 (0.72)	0.405 (0.02)	−0.402 (0.02)	−0.143 (0.43)

*Data are Spearman’s coefficients (*p* values)*.

**Table 4:** Correlation coefficients for the associations of sleep measures with physical and metabolic parameters, dysglycemic group.

	Body mass index	Waist circ.	Body fat (%)	HbA1c	Fasting glucose	2-h Glucose	Glucose areas under the curve	1/Fasting insulin	Insulinogenic index	Oral disposition index
Arousal index, events/h	−0.038 (0.88)	0.009 (0.97)	−0.399 (0.10)	0.046 (0.86)	−0.104 (0.68)	−0.242 (0.35)	−0.164 (0.53)	−0.076 (0.77)	0.147 (0.57)	0.201 (0.44)

Apnea–hypopnea index, events/h	0.172 (0.46)	0.172 (0.46)	−0.088 (0.70)	−0.020 (0.93)	−0.118 (0.61)	0.083 (0.73)	0.170 (0.47)	0.340 (0.17)	−0.171 (0.50)	0.059 (0.82)

Nadir SpO_2_, %	−0.225 (0.33)	−0.331 (0.14)	0.047 (0.84)	−0.006 (0.98)	0.031 (0.89)	−0.169 (0.48)	−0.268 (0.25)	−0.369 (0.13)	0.380 (0.12)	0.157 (0.53)

*Data are Spearman’s coefficients (*p* values)*.

## Discussion

In this pilot study, we evaluated PSG-measured sleep-disordered breathing in obese adolescents with a range of glucose tolerance. The median AHI in this study was 2.2 events/h, and the majority of participants had an AHI less than 5 events/h, which is the threshold for OSA in adults (18). Although participants with dysglycemia tended to have higher AHIs (median 3.2, 2.2, and 1.6 events/h for T2D, prediabetes, and NGT, respectively), there was not a linear relationship between measures of glycemia and AHI. The percentage of participants meeting criteria for any degree of OSA (AHI greater than or equal to 1 events/h) was 66.7% in those with normal glycemia versus 76.2% in those with dysglycemia, although this did not meet criteria for statistical significance (*p* = 0.07). Individuals in each category of glucose tolerance had PSG evaluations consistent with OSA, with wide variability. Higher BMI, higher waist circumference, and greater insulin resistance (lower inverse fasting insulin) were associated with lower nadir SpO_2_ among participants with NGT. Although our hypothesis that youth with dysglycemia would have higher AHI than obese youth with NGT was not verified in this study, our results suggest that a study with a greater proportion of youth with dysglycemia and T2D is warranted and should be pursued through a larger network of clinical sites.

An association with nocturnal hypoxia and hyperglycemia has been shown in adult T2D studies, where greater levels of oxygen desaturation or even relatively mild intermittent hypoxemia were associated with poorer glycemic control (19–23). Also, improving glycemic control in adults with T2D *via* a pharmacological intervention has been associated with a decrease in nocturnal oxygen desaturation (24). The mechanisms of this complex relationship are not yet completely understood, but central, autonomic, and inflammatory mechanisms have been implicated (25–27). Rodent studies have shown that circulating catecholamines act upon α-adrenoreceptors, leading to hyperglycemia and glucose intolerance when intermittent hypoxia is imposed (28).

Pediatric studies have shown that OSA measured by PSG is associated with markers of insulin resistance as measured by fasting insulin and glucose (HOMA-IR) (29, 30), OGTT (10), and intravenous glucose tolerance testing (31). However, pediatric studies examining the relationship between OSA and dysglycemia have had mixed results. Verhulst et al. showed the mean nocturnal SpO_2_ was independently correlated with OGTT glucose area under the curve in lean and obese youth aged 6–16 years (32). However, in the Cleveland Children’s Sleep and Health Study, oxygen desaturation was not associated with OGTT-stimulated glucose concentrations in a community-based cohort of lean and obese adolescents (*n* = 270; mean age 13.7 years; 41% with BMI ≥85th percentile) (30). The fact that most of the participants were not obese in this previous study may contribute to the different outcomes (30). de Sousa et al. compared PSG variables and OGTT-derived measures of glucose tolerance in obese white adolescents with polycystic ovary syndrome (PCOS, *n* = 31) to those of healthy control females (*n* = 19) (33). This study found a positive correlation between AHI and HOMA-IR (*r* = 0.21, *p* = 0.01), but no association between PSG parameters and OGTT glucose measures. The same group studied changes in PSG variables and OGTT glucose measures in obese adolescents treated for PCOS longitudinally (*n* = 15, mean age 15.3 ± 1.2 y, 28 ± 6 months between evaluations) (34). Body composition, PSG variables, and OGTT-derived measures of glucose metabolism were unchanged from baseline. Koren et al. also found no association between measures of sleep-disordered breathing and OGTT measures (35). Similar to our study, participants were all obese (*n* = 62, mean age 14.4 years, range 8–17.5; mean BMI 37 kg/m^2^) with a range of glucose tolerance. The age range was broader in this previous study, and the range of minimum SaO_2_ values was greater in our study.

Shalitin et al. evaluated obese children and adolescents with and without T2D (*n* = 11, *n* = 30, respectively) with PSG and fasting laboratory tests (6). There were no between-group differences in AHI >5/h. The percentage of participants with AHI >5/h was 45% in participants with T2D, 25% in obese participants with IGT, and 18% in obese participants with NGT. Similarly, our study found: AHI >5/h was 43% in participants with dysglycemia and 19% in obese participants without dysglycemia. In both studies, participants with dysglycemia were more likely to have AHI meeting the criteria for OSA; however, between-group differences were not statistically significant in either study. Both studies lacked sufficient participants to adequately test the hypothesis that OSA occurs more frequently in obese adolescents with dysglycemia. It may be that obese youth are at risk for both sleep-disordered breathing and T2D, but the associations are weaker in youth than in adults. It could also be that the pathophysiology linking T2D and hypoxia is much more prevalent in older adults as compared with adolescents. A larger study in adolescents will be required to determine whether or not these speculations are accurate.

This study has limitations that warrant consideration. Participants who reported snoring were recruited from obesity referral clinics, limiting the generalizability to the broader adolescent population. Participants treated with either metformin and/or lifestyle recommendations were eligible, after discontinuing treatment for a short period of time. Previous treatment may have influenced findings in this study. The study was performed in two locations due to the principal investigator relocating, and PSGs were read and evaluated by two sleep investigators without cross-calibrations. However, the same OGTT and PSG protocol was utilized in both locations. To minimize participant burden and enhance enrollment and participation, we opted for OGTT evaluations in lieu of more invasive glucose clamp studies. Measures often performed in adult populations, including neck circumference and level of crowding of oropharynx, were not performed. Sleep data including the percentage of time spent in REM and slow wave sleep and the percent of sleep time below 90% oxygen saturation were not uniformly available. Also, children with a history of tonsillectomy, which is a treatment for OSA in children, were not excluded. The data were not normally distributed, which was expected for glucose measures given the objective of the study. However, considering the preliminary nature of this investigation, we included all of the available data from all participants. There was a predominance of females in the dysglycemia group, as dysglycemia is more commonly occurs in female versus male adolescents. As males are more likely to have sleep-disordered breathing, evidence of sleep-disordered breathing in obese adolescents with dysglycemia could be underestimated in this setting. It must be noted that a larger sample size is required to provide a definitive answer to the question of whether or not OSA is related to dysglycemia in adolescents. Given the nature of this pilot study, an adequate sample-size calculation was not performed at the onset. Perhaps with a larger study, including more participants with T2D, sleep-disordered breathing would be seen more predominantly.

Strengths of the study include a strong rationale, given the epidemiological prevalence of OSA in obese adults and the high percentage of adults with both T2D and OSA, and the lack of previous studies in obese adolescents. The inclusion of only obese pubertal patients aged 12 years and up is of particular value given the differences in glucose metabolism in pre-pubertal and adolescent patients (7). The mean age of diagnosis of T2D in youth is 14 years (36); thus, our cohort is representative of an appropriate population for evaluation of diabetes risk markers.

In conclusion, although we did not confirm the hypothesis that obese youth with dysglycemia have more characteristics of OSA than obese youth with NGT in this pilot study, we have further added to the literature on adolescent obesity, dysglycemia, and sleep characteristics. A larger study involving a network of clinical research sites is warranted to further understand the relationship between sleep-disordered breathing and dysglycemia in obese adolescents, especially since two pediatric studies have now shown a greater proportion of OSA in obese youth with dysglycemia versus NGT. Further study with a larger proportion of youth with prediabetes and T2D will also be necessary to determine whether evaluation for sleep-disordered breathing is uniformly warranted.

## Ethics Statement

This study was approved by the University of Pittsburgh and Indiana University Institutional Review Boards and performed in the Pediatric Clinical and Translational Research Center (Children’s Hospital of Pittsburgh) and the Indiana Clinical Research Center with collaboration from the clinical sleep centers at these institutions. All participants and their parent (guardian) gave informed consent before participating.

## Author Contributions

TH wrote the draft of the manuscript. SW collected data and performed research procedures. HJ interpreted data. SC interpreted data. KM provided key manuscript edits and mentoring. SA provided key manuscript edits and mentoring. No honorarium, grant, or other form of payment was given to anyone to produce the manuscript. There are no prior publications or current submissions to other journals with overlapping information.

## Conflict of Interest Statement

The authors have no conflicts of interest to disclose. The study sponsor(s) had no role in (1) study design; (2) the collection, analysis, and interpretation of data; (3) the writing of the report; or (4) the decision to submit the paper for publication. TH wrote the first draft of the manuscript. No honorarium, grant, or other form of payment was given to anyone to produce the manuscript. The reviewer KO and handling Editor declared their shared affiliation.
